# Recent developments in using the molecular decay dating method: a review

**DOI:** 10.1111/nyas.14560

**Published:** 2021-01-14

**Authors:** Johannes Tintner

**Affiliations:** ^1^ Department of Material Sciences and Process Engineering Institute of Physics and Materials Science, University of Natural Resources and Life Sciences Vienna Vienna Austria

**Keywords:** taphonomy, organic matter, archaeometry, FTIR spectroscopy

## Abstract

The dating of organic findings is a fundamental task for many scientific fields. Radiocarbon dating is currently the most commonly used method. For wood, dendrochronology is another state‐of‐the‐art method. Both methods suffer from systematic restrictions, leading to samples that have not yet been able to be dated. Molecular changes over time are reported for many materials under different preservation conditions. Many of them are intrinsically monotonous. These monotonous molecular decay (MD) patterns can be understood as clocks that start at the time when a given molecule was formed. Factors that influence these clocks include input material composition and preservation conditions. Different wood species, degrees of pyrolysis, and pretreatments lead to different prediction models. Preservation conditions might change the speed of a given clock and lead to different prediction models. Currently published models for predicting the age of wood, paper, and parchment depend on infrared spectroscopy. In contrast to radiocarbon dating, dating via MD does not comprise a single methodology. Some clocks may deliver less precise results than the others. Ultimately, developing a completely different, new dating strategy‐such as MD dating–will help to bring to light a treasure trove of information hidden in the darkness of organic findings.

## Graphical abstract

This review focuses on molecular changes over time. Having been discussed since the 1960s, the variety of molecular clocks includes the change in amino acids in the primary structure of a protein over time. Molecular decay (MD) has a mainly monotonous function, a prerequisite to serving as such a clock. In addition, such clocks need a defined starting point and a defined decay function. In contrast to the decay of alpha and beta emitters, MD is influenced by several environmental factors indicated by preservation conditions. The MD method has the potential to be used for different organic materials and different scientific fields.

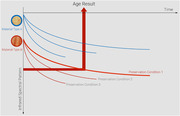

## Introduction

Without knowledge of age, findings cannot be set in an archaeological, historical, or forensic context. Age is the most important question posed whenever an unknown object is found. Dating can be done by relative or chronometric methods, and they can be numerical or not. Relative methods are based on stratigraphic patterns at the excavation site, whereas chronometric methods generally use a clock driven by the radiometric decay of alpha and beta emitters.^1^ Some methods were first considered as chronometric methods, while after some time this assignment was put into a different perspective. A chronometer must meet three requirements: (1) it must be a constant process that affects a given material (e.g., a molecule); (2) the material used for dating must be considered to be a closed system, that is, there is no exchange of matter and energy between the system and surrounding systems; and (3) the rate of change must not be affected by environmental factors. The most prominent example—radiocarbon dating—can be applied to organic matter and uses the decay of ^14^C (see Refs. 2 and 3). The unique pattern of annual climatic variation is fixed in growth patterns found in tree rings or growth lines of mussel casks. Connecting the information from many individuals of different ages creates chronologies that can be used to date samples of unknown age. The corresponding methods are referred to as dendro‐^4^, ^5^ or sclerochronology.^6^ Amino acid dating uses the trend of racemization, turning the ratio of d and l configurations of amino acids from almost zero to an equilibrium of about one.^7^, ^8^ In contrast to dendrochronology or radiocarbon dating, amino acid dating is affected by several environmental preservation conditions.

This review focuses on molecular changes over time. “Molecular clocks” have been discussed since the 1960s, and refers to the replacement of amino acids in the primary structure of a protein by other amino acids. These clocks have been calibrated for mitochondrial proteins.^9^ They have aided dating of the time of divergence of hominid primates from other mammals.^10^ Molecular decay (MD) has a mainly monotonous function, a prerequisite to serving as a clock (point (1) above). Such clocks need a defined starting point and a defined decay function. Contrary to the decay of alpha and beta emitters, MD is influenced by several environmental factors indicated by preservation conditions. These preservation conditions have to provide at least approximate closed conditions (point (2) above). The starting point can also vary considerably. Therefore, there is no unique model that can be used for all kinds of organic matter, as for each type of material there is a whole array of clocks. Approximate knowledge about the starting point and preservation conditions are necessary to date the new samples. Environmental factors must be stable enough to be defined as stable and not changing fluently (point (3) above). A few first cases have already been published (for some wood species,^11^, ^12^ paper,^13^ and parchments^14^), but further input from the scientific community in the future could complete the picture. Ultimately, MD can serve as an independent, innovative dating approach.

## MD dating

**Figure 1:**
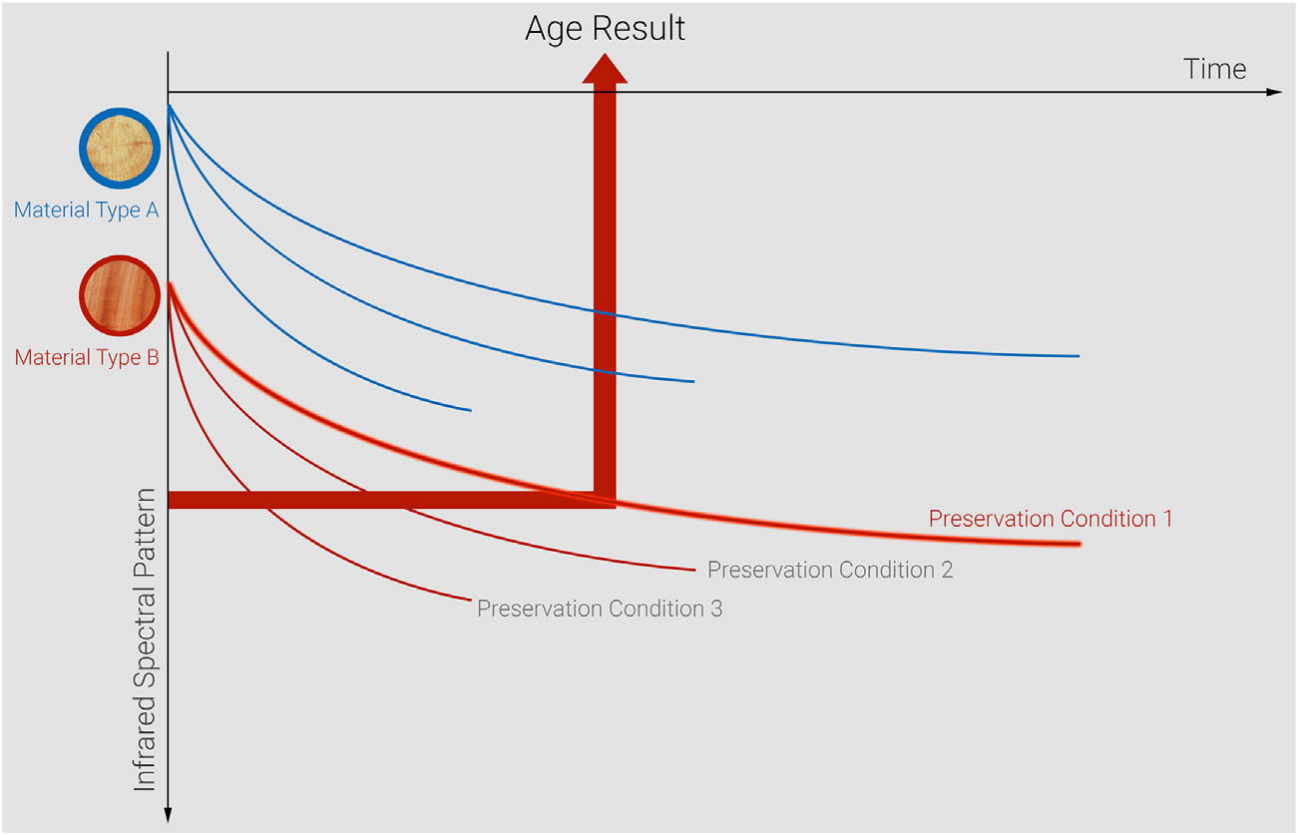
Molecular decay (MD) dating cannot be defined as one tool, but rather comprises many models dependent on specific material types and preservation conditions. Schematic subdivision of dating tools for a certain material (e.g., wood) based on the infrared spectral pattern, material types A and B could represent different species, and preservation conditions 1, 2, and 3 could represent dry, salt, and bog storage; with a known material type (B) and preservation conditions (1), infrared spectral pattern results in the age of the sample (age result).

The following section describes the current status on the path toward dating tools based on MD. Figure 1 displays the general routine of age estimation via MD dating. First, the models of decay for different material types and preservation conditions must be established. The material type and respective preservation condition of an unknown sample must be estimated with considerable references. Then, the spectral pattern is measured, and the position on the curve is elaborated. A key task is the definition of the parent material. It serves as a starting point of the decay function and needs to be defined carefully according to its chemistry. Decay processes must be defined as exactly as possible; however, for a usable model, it is even more important to detect chemical changes precisely rather than to explain these changes in detail. Preservation conditions determine the living conditions for microorganisms responsible for structural decay. Besides the environmental factors, random accessibility and the presence of microorganisms might play a certain role. Several archaeological sites have survived catastrophes, or, to say it less dramatically, extraordinary events that might have given only a limited number of microorganisms the chance of access. The influencing factors need to be identified. It must be checked to which extent they change the decay function. In many cases, countless environmental factors might be suspected to impact MD. Previously published models demonstrate that at least some of them are important; however, they are either of negligible impact or their impact is included in a prediction error that remains at an interestingly low level. A prerequisite for dating is that environmental factors remain stable over deposition time. Incorrect assignments of preservation conditions and/or material type lead to inaccurate predictions. If the age of an artifact is known, it might be possible to reconstruct long‐term environmental conditions. Currently, this possibility remains theoretical.

It is important to apply both an adequate analytical tool and statistical modeling. The selection of proper analytical tools depends a great deal on the exact question. The description of MD in organic matter can be performed by numerous methods; these include both destructive and nondestructive methods. Representative members of the group of destructive methods are thermal analyses, pyrolysis gas chromatography, and time‐of‐flight secondary ion mass spectrometry. Trojanowicz^15^ stressed the advantages of some spectroscopic methods for a nondestructive analysis of organic molecules in archaeometry, especially reflectance UV‐vis, Raman, Fourier transform infrared (FTIR), nuclear magnetic resonance (NMR) spectroscopy, and fluorescence microscopy. These methods received increased attention as sophisticated chemometric methods became available. FTIR spectroscopy, in particular, combines the advantages of relatively low costs and high speed. Molecular changes of organic matter are widely detectable, and huge sample sets can be measured with acceptable effort. Because of that, it is not astonishing that all currently published dating models used FTIR as an analytical tool.

**Figure 2:**
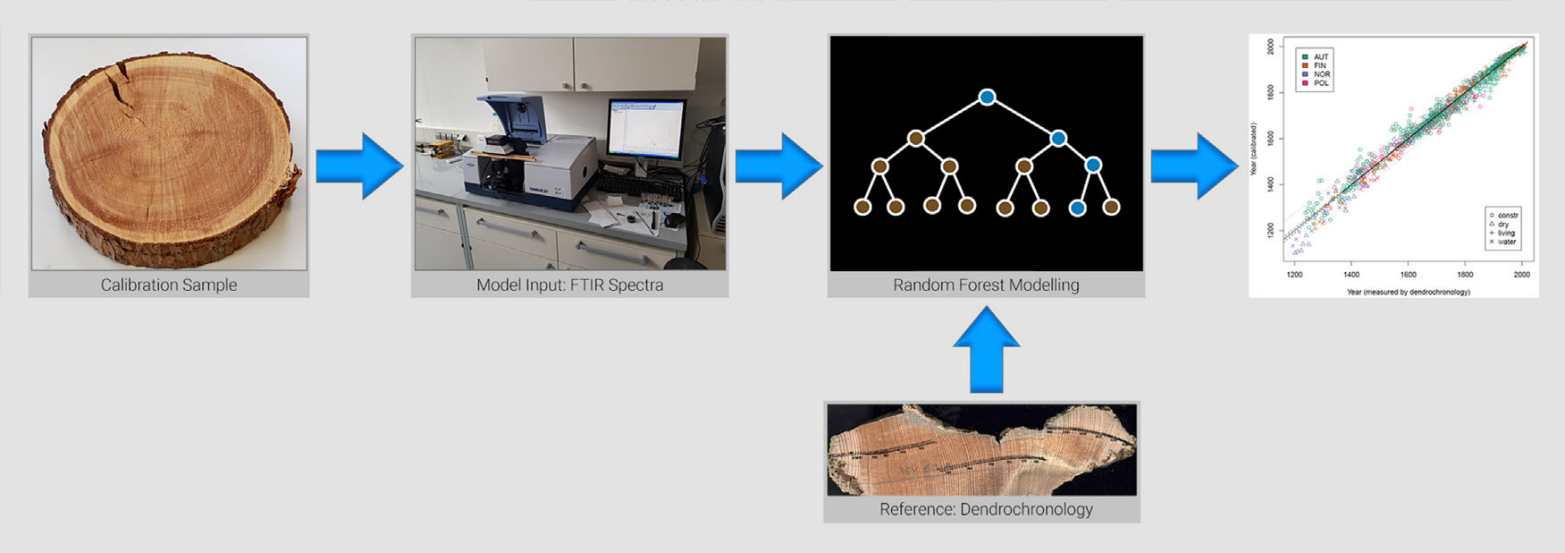
Example procedure to establish an MD dating tool. The example shows the MD dating tool for pinewood.^12^

The statistical evaluation has to handle the fundamental data situation. The entire spectrum or at least its selected features will be linked to age by training on a set of reference data. A prediction interval will be provided by the model describing its precision. Among chemometric methods, partial least squares (PLS) regression is commonly applied to predict continuous traits out of multivariate analytical data sets. It has also been applied to dating models (e.g., Refs. 13, 14, and 16). The field of machine learning bore several techniques with completely different statistical approaches. They emerged in very different fields of application with huge data sets. Roughly, they can be separated into supervised and unsupervised techniques. Representative members of supervised learning are regularization methods for prediction and classification, additive and tree‐based models, neural networks, and support vector machines. Representative members of unsupervised learning are clustering algorithms, self‐organizing maps, principal and independent component analysis, and multidimensional scaling. Recently, random forests have been applied to dating models of wood as an example of a tree‐based model.^11^, ^12^ Figure 2 displays the general pattern regarding how MD dating tools are created on the basis of the pinewood model.^12^

An interesting aspect of different MD dating models for different preservation conditions is the possibility to change the direction of the arrow in Figure 1. Material type, preservation condition, and age are linked by these models. When material type and preservation conditions are known, age can be predicted—this represents the normal case of dating. If the material type and age are known, long‐term preservation conditions can be returned by molecular constitution. In Figure 1, this would mean that an artifact of, for example, material type B is measured by FTIR spectroscopy. Age is known, and the fitting preservation conditions can, therefore, be extracted based on the infrared (IR) spectral pattern. It must be clarified that currently, no such applications are published as only for wood, a limited set of different preservation conditions has been tested using MD dating tools. Therefore, future work will have to prove whether such reconstructions of preservation conditions stay only extremely generic or can deliver a more detailed picture. Information on long‐term preservation conditions cannot be answered by many other methods and could lead to completely new archaeometric research questions.

The following subsection describes the current status of MD dating with respect to Table 1 for different organic materials (e.g., wood, bark, straw, and paper).

**Table 1:** Factors of subdivision leading to different models for MD dating tools

Parent material	Material subdivision	Decay process subdivision	Already existing model approaches	Applications
Lignocellulosic material				
Wood	Species^15^ and part of the wood (trunk, branch,^16^ and root)	Preservation in soil: temperature, pH, and moisture content^17^, ^18^	Prediction based on random forest models^9^, ^10^	Building history and archaeology^19^
Bark	Species^20^	Preservation corresponding to the wood	Separation based on PCA^21^	Archaeology, mobile art, and cultural objects
Straw	Straw part (nodes, culm, and awn)^14^	Preservation in clay and distance in bricks to the surface	Prediction based on PLS regression^14^	Adobe constructions, clay plasters, and archaeology
Paper	Paper‐making process and surface stabilization	Preservation in archives: temperature, UV light, and pH^22^	Prediction based on PLS regression^11^	Archives
Charcoal	Pyrolysis conditions and feedstock^23^	Preservation in soil	Separation based on PCA^24^, ^25^	Archaeology, paleoecology, landscape, and history ^26^, ^27^
Proteinaceous material				
Keratin‐containing material				
Hair	Species and pretreatment	Preservation conditions: temperature, pH, and plaster matrix^28^, ^29^, ^30^		Archaeology, building history,^31^ and forensics
Collagen‐containing material				
Bone (antler)	Bone type, species, and pretreatment like boiling^32^, ^33^	Preservation in soil and tombs^34^		Archaeology, forensics, and archives
Skin (leather and parchment)	Species and parchment treatment^35^, ^36^	Preservation in archives and tombs	Prediction based on PLS regression^12^	Archives, archaeology, and forensics
Amber	Amber class^37^	Preservation in soil and archives^38^, ^39^, ^40^, ^41^		Archaeology and art history

### Lignocellulosic material

#### Wood

Wood is the most important organic construction material and has been so from the beginning of humankind up until today. Its relatively high recalcitrance makes it one of the most abundant organic materials in archaeology. The starting points for MD dating tools differ in particular by species. Wood chemistry differs in the main compounds of the lignocellulosic complex and extractives. Differences in the aging processes were recorded between the pine and oak samples from the 16th century.^17^ This shows the need for different dating tools for different tree species. Pizzo *et al*.^44^ presented prediction models for lignin and holocellulose in archaeological waterlogged wood based on FTIR spectra. The results display a clear decrease in acetyl groups in hemicelluloses. The paper is a rare example in which results relating to ash and elm in addition to softwood and oak are presented. Different parts of the tree (trunk, branch, knots, root, etc.) lead to differences in chemical composition^18^ and limit the starting point for dating models. This might lead to different MD dating models. It depends whether chemical differences in the wood chemical structure are located in relevant parts of the FTIR spectra, where aging effects are mirrored. Tintner *et al*.^45^ demonstrated that earlywood and late wood differences are mainly driven by lignin content and, therefore, load on a different component in principal component analysis (PCA) than the age differences over 3500 years. Other wood chemical differences like sapwood/heartwood or compression/tension wood have not been studied in this specific light. Łucejko *et al*.^21^ gave not only a comprehensive review of degradation in archaeological wood but also a compilation of different analytical, especially spectroscopic, methods that are commonly used. Kim^46^ described chemical changes in marine waterlogged wood. The effect of ion exchange altered the elemental composition, leading Mg, K, and Ca to decrease and S and Fe to increase. Wood chemistry was determined by means of wet chemical analyses and FTIR spectra. A relative decrease in holocellulose was paralleled by a relative increase in lignin and extractives. The most important impact on wood decay is microbial degradation. In waterlogged archaeological wood, this degradation is dominated by erosion bacteria and soft rot fungi. The former need less oxygen and, therefore, go deeper into the logs.^47^ There is a significant decrease in decay from outer zones to the core. Speed depends on various factors, the most important of which are environmental conditions regulating oxygen supply and the species of wood. Despite the high complexity, sound areas can be found even after a long time.^48^ Changing conditions, such as those brought on during drainage and foster, decay by white rot fungi.^19^ Generally, degradation follows a common pattern. A remarkable result was found for 300‐year‐old spruce poles from a moat in Copenhagen. FTIR spectra hint at a degradation process comparable with a piece several hundred years older. This might mean exceptional preservation conditions, at least for some time, or the starting material was already anomalous 300 years ago. The section was sampled up to a depth of 2.5 centimeters. Unfortunately, the center (at a depth of 6 cm) was not sampled.^49^ Decay under dry terrestrial storage conditions is a mixture of microbial and nonbiological decay. Even in hot and cold deserts, both degradation types take place.^20^ MD starts at the weakest parts of the lignocellulosic complex—hemicelluloses. The near‐IR (NIR) spectra of two cypress samples (one from a building constructed in the year AD 750 and one built recently) displayed a decrease in bands assigned to hemicelluloses and the amorphous region in cellulose.^50^ Sandak *et al*.^51^ presented a general compilation of reasons in favor of using IR spectroscopy (in this case, NIR) in the archaeometric assessment of archaeological wood. The main advantages are accuracy, simplicity, high speed, and low costs. The results were obtained from five oak samples from waterlogged, air dried, and peat bog environments.

Guyette and Stambaugh^52^ presented a dating model for oak wood buried in sediments. The model is based on wood density and covers about 12,000 years. Prediction quality is rather low, with an uncertainty of around 1000 years. As wood density can be predicted on the basis of FTIR spectra,^53^, ^54^ we can assume that chemical composition comprises the underlying effect that establishes the model for age. Inagaki *et al*.^55^ described chemical changes in waterlogged archaeological cypress wood. They present a model for age prediction based on the NIR spectra of two samples covering 400 years with a prediction quality of about 40 years. Most recently, Tintner *et al*.^11^, ^12^ presented prediction models for five different wood species covering up to 3000 years based on the statistical model approach of random forests. MD was measured using ATR‐FTIR. Some relevant restrictions were made for these models: brittle parts, especially those found in waterlogged wood, were not considered. For the presented models regarding subfossil wood, only intact pieces without severe microbial decay can be used for these models. The second reason for model failure affected the area nearest to the surface in a construction wood as a result of increased access to oxygen.^11^ The latter excludes all thin‐walled painted panels or small sculptures. Many wooden artifacts in the museums and archives cannot be considered by this current model.

Summarizing the current status for wood, it is obvious that different species or genera lead to different models. It is also rather well known that MD takes place in the wood. The influencing factors that subdivide the dating models are different preservation conditions, but it seems that not all preservation conditions must be separated into different models. Both mathematical approaches, PLS and random forests, were successfully applied, although random forest seems to result in better models and is the favored method.

#### Bark

There are other lignocellulosic materials less recalcitrant and, therefore, less common in archaeology. Only specific preservation conditions allow the bark to survive over centuries or millennia. Long‐term survival of such materials is documented from ice sites in the European Alps.^56^ Pacific barkcloth from the 18th and 19th centuries can be separated according to different tree species. FTIR spectra also revealed evidence of a structural decrease in acetyl groups in hemicelluloses.^22^ This decrease was the dominating taphonomic process detected using ATR‐FTIR in the Bronze Age bark found at a salt mine in Hallstatt, Upper Austria. PCA was applied to differentiate recent and prehistoric bark.^23^

#### Straw

Straw is not a very common finding in an archaeological context, whereas it is quite common in clay constructions as an amendment for reinforcement.^57^ Tintner *et al*.^16^ presented a preliminary prediction model for age based on the MD of straw in clay bricks and plasters measured by using FTIR spectroscopy based on 14 historic objects spanning about 480 years with a prediction error of 93 years.

#### Paper

Paper is a lignocellulosic material mainly found in archives. It was introduced to Europe in the 12th century by the Arabs, who obtained the technology from the Chinese much earlier. Paper can be chemically differentiated according to processing techniques, especially surface stabilization. During the mid‐19th century, several technological changes make it difficult to combine historical and recent papers into a single model. The main degradation reactions are hydrolysis of cellulose, oxidation of lignin, and thermal degradation.^58^, ^59^ Cellulose aging and lignin degradation (yellowing) in newsprint can be separated using FTIR spectroscopy.^24^

Trafela *et al*.^13^ present a dating tool for historical paper based on IR spectra. One model was established using 204 recent samples dating between now and AD 1850, and another one on 28 samples from AD 1850 to AD 1650. Prediction qualities are very good with around 8 years for both models. An even more complex material problem was assessed by Martins *et al*,^60^ who provided dating tools for fiber‐based gelatin silver prints with very good prediction qualities. The differentiation of the starting points can be assigned to geographical origin and manufacturing process. Comparable results were found for chromogenic color photographs.^61^

### Charcoal

Charcoal as the solid residue of pyrolytic processes is a common residue in archaeological excavations. But investigations in the field of paleoecology and paleogeography also make use of charcoal residues in soils.^28^ Conedera *et al*.^29^ described the importance of past fire regimes. Its reconstruction is often based on charcoal assemblages in soils. Currently, radiocarbon dating is the state‐of‐the‐art method for such reconstruction analyses.^62^ An important issue of radiocarbon dating and dendrochronology is the so‐called “old wood problem,” meaning that both methods date the year when the tree ring was created by the tree.^63^ The starting point of the clock using chemical changes in charcoal is the time of pyrolysis. Influence factors on charcoal chemistry and, therefore, subdividing factors for dating models are pyrolysis conditions and feedstock for the starting point and environmental preservation conditions for the decay function.^25^ Pyrolysis temperature changes charcoal properties in a specific way. A predictive model for the degree of carbonization measured using FTIR spectroscopy has been presented.^64^ Théry‐Parisot *et al*.^65^ gave a comprehensive overview of taphonomic processes of charcoal in an archaeological context. The main chemical process that takes place is oxidation, leading to an increase in the O:C ratio and changes in the surface charge from positive to negative.^66^, ^67^ Smidt *et al*.^27^ used the characteristic changes in FTIR spectra and results of simultaneous thermal analyses (STAs) to distinguish charcoal residues from different periods (recent, Modern Period, Medieval Period, and Bronze Age) based on PCA. Furthermore, they were able to distinguish medieval kiln charcoal from combustion residues. This pattern can be altered by exceptional pyrolysis and/or preservation conditions.^26^ The stability of biochar in soils can be estimated using the O:C molar ratio^68^ or the behavior in STA.^69^

### Proteinaceous materials

The third important group of organic matter in heritage science are proteinaceous materials. Keratin‐containing hair and collagen‐containing skin are rather rare and limited to exceptional preservation conditions, whereas collagen‐containing bones are rather common due to the recalcitrant inorganic matrix. Skin has a special relevance as parchment documented in archives. The spatial discrimination of different tissues (nail, skin, and bone) in ancient mummies has been worked out by a nondestructive, portable NMR.^70^

#### Hair

Hair is a surprisingly durable material. It is difficult to find it in archaeological excavations, but it contains highly persistent proteins. Bonnichsen *et al*.^71^ reported a 9800‐year‐old strand of sheep hair found inside a cave under dry preservation conditions. Keratin from hair and feathers is degraded in the soil system only by a few types of enzymes.^72^, ^73^ The pH value of preservation conditions has a proven effect on the thermal stability of hard alpha‐keratin from hair.^30^ Kennedy *et al*.^31^ reported on the oxidation of cysteine into cysteic acid after an acidic treatment as leading to unfavorable properties for its use in lime plasters. Its recalcitrance in historic plasters has been documented.^32^ Historic hair is found in the archaeological context with mummies and might be often overseen in excavations. Not only textiles and tapestries but also plasters contain hair.^33^, ^74^, ^75^, ^76^ FTIR spectroscopy proved the oxidation of disulfide bonds as a matter of oxidation in the wool of historic Tudor tapestries.^74^ Different conservation oils and ointments lead to different status of conservation statuses in terms of surface and mechanical properties of Copt mummies from the first Christian era.^75^ Owing to the limited number of artifacts, there is still no concise picture, but the individual chemical composition of materials from different species leads to diverse degradation pathways. Preservation conditions such as pH, temperature, moisture content, and oxygen access will also affect MD.

### Collagen‐containing materials

Collagen is the most abundant protein on Earth and can be found in many structural animal components like skin or bones. But tendons, fish scales, antlers, and dental enamel also contain collagen. The FTIR analysis is among the most powerful and useful tools for the assessment of collagen from very different origins.^77^

#### Bone

Turner‐Walker^78^ provides a comprehensive review of the chemical and physical constitution of bones and teeth and their chemical and microbial degradation. He worked out the relevance of various factors, including soil hydrology, temperature, and pH, that influence the speed of degradation. The two mechanisms that have been identified as the main degradation paths are bacterial degradation and chemical hydrolysis of bone collagen. Dobberstein *et al*.^79^ clarified the diagenesis of collagen in comparison to osteocalcin, the second most common bone protein. The fate and diagenesis of osteocalcin has been described from archaeological material.^80^ Collagen follows a sigmoidal loss with a stable amino acid profile until a collagen yield below 1 percent. Special preservation conditions of chicken bones in salt (halite) resulted in ion exchange and a strong increase in bone mineral content within several weeks; Mg, Ca, and P contents decreased, and Na increased.^81^ Synchrotron radiation FTIR spectroscopy has proven to be a powerful tool to study fossil bone alterations at the microscale, as shown by two bone samples with ages of 15 and 60 kiloamperes.^82^ There is even evidence of collagen preserved in a 195‐million‐year‐old sauropodomorph dinosaur.^83^ Surmik *et al*.^36^ provided evidence of collagen in bones of around 250‐million‐year‐old marine reptiles using multiple spectroscopic methods. The tissues were preserved well in the iron oxide–rich sediment. An in‐depth analysis of degradation was performed by means of synchrotron radiation–based FTIR spectroscopy applied on a well‐preserved 5000‐year‐old archaeological bone.^84^ Only small areas revealed degradation indicators as reducible/nonreducible cross‐links (1690/1660 cm^−1^) and, to a lesser extent, the random coils/α‐helix ratio (1645/1660 cm^−1^). This work is linked to another work that described the different molecular components contributing to the broad amide I‐band between 1700 and 1600 cm^−1^ (Ref. 85). Nielsen‐Marsh *et al*.^35^ gave a comprehensive picture of structural changes in archaeological bones based on the results of STAs. They also stressed the effect of cooking on degradation status, indicating thermal age as a measure of degradation in STA results. Structural damages to bones in terms of porosity and protein content have been shown to depend on burial environments.^34^

#### Skin

Aging processes in the skin already begin during the body's lifetime. In addition to internal aging effects, the exposure to air and especially UV light fosters these processes, making the skin less elastic, wrinkled, stiffened, and less able to recoil.^86^ Different enzymes are reported to degrade collagen in the extracellular matrix of skin.^87^ Brandt *et al*.^88^ demonstrated the difficulties of microscopic species determination of archaeological skin objects preserved in bogs. They proposed mass spectrometry‐based peptide sequencing as a valid method. Before that, DNA was the only common carrier of this information.^89^ Salt preservation results in favorable conditions for halophilic microorganisms. Enquahone *et al*.^90^ reported specific red heat damages on salted hide and skin.

A number of skin artifacts are in the form of leather or parchment. Kennedy and Wess^91^ compiled deterioration processes in parchment—oxidation, hydrolysis, and gelatinization—that lead to collagen molecules breaking into smaller peptides, the loss of their triple‐helical structure, and the characteristic hierarchical organization. Untreated leather from archaeological excavations becomes lightly colored on the surface and hard and stiff when allowed to dry.^92^ Natural aging and the effects of alkaline and acidic treatments of leather and parchment have been studied by thermal analyses.^37^ ATR‐FTIR has been used to detect gelatinization and calcium stearate formation in leather book covers from the 17th century.^93^ Orlita^94^ compiled a review on the microbial deterioration of leather and its control. Microbial succession has been proposed as leading to purple spot deterioration of parchment.^95^ Red heat degradation of chrome‐tanned leathers also depends on the same starting organism.^96^ Alvarez *et al*.^97^ demonstrated the information content of an optical fingerprint of historical parchments. They were able to discriminate not only among different species but also among different manufacturers. They also reported material modifications due to aging. A comparison of manufacturing techniques and salt incrustations was performed using spectroscopic methods.^38^ Different tannins in historic leathers from the 19th century have been identified using IR spectroscopy.^98^, ^99^ Možir *et al*.^14^ presented a PLS regression dating model for parchment based on oxidative degradation measured by IR spectroscopy. The model includes 185 historical objects covering 600 years, from around AD 1200 to around AD 1800, with a prediction error of 72 years.

Summarizing the current status for parchment, leather, and skin: the starting points for dating models are different species and, especially, different manufacturing techniques. MD here, as for the other materials highlighted above, is influenced by preservation conditions. Apart from all these influencing factors, a dating model for parchments proves the applicability of MD for dating of such materials.

### Amber

Different classes of fossil resin can be discerned: polylabdanoid, cadinene‐based, polystyrene, cedrane sesquiterpenoid, and abietane/pimarane diterpenoids.^39^ Copal can be seen as the more susceptible (and mostly younger) relative of amber.^100^ Taphonomic processes comprise oxidation and metal carboxylate formation. The class of amber played a defining role in determining the chemical pathways of degradation. In particular, photodegradation affects amber significantly but also temperature, oxygen, relative humidity, and pH play relevant roles, leading to depolymerization and oxidation.^41^, ^42^, ^43^ Generally, amber must be seen as quite susceptible to degradation.^40^ Analytical differences can also be detected for different amber provenances, although the differences are rather small.^101^ Spectroscopic methods (NMR and FTIR) were used as analytical tools.^102^, ^103^ Drzewicz *et al*.^104^ presented a comprehensive review about the pros and cons of different analytical methods to assess amber, including spectroscopic methods (FTIR, Raman, fluorescence, and NMR), chromatography, and mass spectrometry, and others (STA, X‐ray diffraction, and microhardness).

## Unresolved questions and benefits

MD for dating purposes is a promising approach. The first models demonstrate significant potential. The greatest strength of the approach is the fact that MD can be measured comparatively easily and cheaply (e.g., by means of IR spectroscopy). The rising number of multivariate statistical methods available, especially in the field of machine learning, can even help in the establishment of the models. Molecular changes can be unveiled that were previously hidden. Easy and cheap, IR spectroscopy allows the measurement of huge sample sets. Especially in the case of recalcitrant materials (charcoal, wood, and bones), a vast amount of material can be found in archives and collections. Sample sets with hundreds or thousands of samples will result in well‐validated models. The easy and cheap measurements will provide the opportunity to answer questions on the application of the models that have not been formulated thus far. Huge sample sets will allow estimating the spatial heterogeneity of the age of findings in the full range. The information contained in the artifacts can be used in more detail. Currently, such full range assessments are rare owing to financial limits. Restrictions are the broad spectrum of materials and preservation conditions. Only for samples where these data are known could dating based on these models be done. Dating can only be performed if prediction tools are already established for respective conditions. Valid prediction tools demand a structural understanding of the MD behind the model. In particular, the influence of different preservation conditions is often not fully understood but could be used as an additional beneficial piece of information. A crucial point is the understanding of extremophilic microorganisms. It should be clarified explicitly that heterogeneous preservation conditions crossed with random accessibility regarding microbial attack limit the prediction quality.

## Competing interests

The author declares no competing interests.
